# Integrative Soil Application of Humic Acid and Foliar Plant Growth Stimulants Improves Soil Properties and Wheat Yield and Quality in Nutrient-Poor Sandy Soil of a Semiarid Region

**DOI:** 10.1007/s42729-022-00851-7

**Published:** 2022-05-02

**Authors:** Ayman M. M. Abou Tahoun, Moamen M. Abou El-Enin, Ahmed G. Mancy, Mohamed H. Sheta, Ahmed Shaaban

**Affiliations:** 1 Agronomy Department, Faculty of Agriculture, Al-Azhar University, Cairo, 11884 Egypt; 2 Soils and Water Department, Faculty of Agriculture, Al-Azhar University, Cairo, 11884 Egypt; 3 Agronomy Department, Faculty of Agriculture, Fayoum University, Fayoum, 63514 Egypt

**Keywords:** Degraded sandy soil, *Triticum aestivum* L., Grain yield and quality, Organic amendments, Aggregate size distribution, Dry mean weight-diameter

## Abstract

Sandy soils (containing > 50% sand) are widely distributed worldwide and are characterized by their poor structure, low organic matter, weak hydraulic and nutritional properties, and low crop productivity. Using a 2-year pot experiment, in this study, we investigated the effects of humic acid (HA) as a soil amendment and study two plant growth stimulants (PGSs), zinc oxide nanoparticles (ZnONPs), and L-tryptophan (L-TRP), as a foliar application on wheat grown in nutrient-poor sandy soil. Three HA rates (0 (HA_0_), 0.2 (HA_0.2_), and 0.4 (HA_0.4_) g kg^−1^ soil) and five PGS levels [control, 50 mg l^−1^ (ZnONPs_50_), 100 mg l^−1^ (ZnONPs_100_), 0.25 mmol l^−1^ (L-TRP_0.25_), and 0.5 mmol l^−1^ (L-TRP_0.5_)] were used. The soil hydro-physico-chemical properties, morpho-physiological responses, yield, and quality were measured. HA addition amended the soil structure by allowing rapid macroaggregate formation, decreasing bulk density and pH, and increasing porosity and electrical conductivity, thereby improving soil hydraulic properties. HA_0.2_ and HA_0.4_ additions improved growth, yield components, and grain minerals, resulting in higher grain yield by 28.3–54.4%, grain protein by 10.2–13.4%, wet gluten by 18.2–23.3%, and dry gluten by 23.5–29.5%, respectively, than HA_0_. Foliar application of ZnONPs or L-TRP, especially at higher concentrations compared to the control, noticeably recorded the same positive results as HA treatments. The best results were achieved through the integration of HA_0.4_ + ZnONPs_100_ or L-TRP_0.5_ to the tested nutrient-poor sandy soil. The interactive application of HA_0.4_ + ZnONPs_100_ or L-TRP_0.5_ and the use of mineral fertilizer, which is considered a surplus point in permaculture, can be recommended for sustainable wheat production in nutrient-poor sandy soil.

## Introduction

Food demand is constantly increasing due to rapid global population growth, which is projected to reach 10 billion by 2050 and brings shifting consumption patterns and ongoing climate change challenges (United Nations 2017). Cereal crops, including wheat, are considered major staple foods in many regions of the world, and they supply more than 50% of the total daily human calorie requirements (World Health Organization 2003). Owing to its high adaptability across a variety of environments (Reynolds et al. 2012), wheat (*Triticum aestivum* L.) is the world’s second most cultivated cereal crop, which supplies approximately 20% of the total world cereal requirements (Jones et al. 2015) with an estimated production area of approximately 200 million hectares (FAOSTAT 2019). Wheat grains are rich in carbohydrates, vitamins, minerals, fats, and dietary fiber and have a higher protein content than other crop grains (Poole et al. 2021). Furthermore, the vital wheat gluten percentage, which ranges from 30 to 35% (Li et al. 2022), plays an important role in wheat-baked food products by helping to standardize dough traits and improve baked product volume (Schopf et al. 2021).

Sandy loam soil, a poor plant growth medium, is considered a serious agricultural challenge for food security in many of the world’s arid and semiarid regions (Zhang et al. 2020). Soil texture is highly reliant on the relevant parent material that plays a key role in forming variations in organic carbon (OC) pools by influencing soil organic matter (OM) stabilization through organo-mineral interactions and the dynamics of aggregation and carbon (C) sequestration (Angst et al. 2018). Fertility and water-holding capacity (WHC), for this kind of soil, are mostly low due to the poor physical structure resulting from a very low OM level (Zhang et al. 2017) and relatively high pH that hinders nutrient absorption and limits mobility and bioavailability of nutrients (Barker and Pilbeam 2015) and consequently a yield reduction. Soil addition of humic acid (HA), as a natural substance for modifying the physical and chemical properties of defective sandy loam soil, could be an effective and viable practice for increasing crop yield and quality (Khan et al. 2018; Nasiroleslami et al. 2021). HA is the principal component of humic substances (HSs), which are the main organic constituent in soils that originate mainly from the biodegradation (i.e., humification) of dead plants and animal residues by microbial activities (Stevenson 1994). Owing to the diverse reactive surface of HA functional groups, such as -COO, -OH, and -NH_2_ (Wang et al. 2019), HA has a high tendency to form stable colloidal aggregates that provide potential binding sites for chelating macro- and micro-nutrients and consequently improve soil fertility. Of the HSs, HA is a dissoluble material at a basic pH that can modulate soil properties and plant nutrient bioavailability and consequently boost photosynthetic activity (Ding et al. 2021) and crop productivity in sandy loam soil (Khan et al. 2018). Soil-applied HA can have beneficial direct physio-biochemical effects on plants, such as increasing the membrane permeability of plant roots (Muscolo et al. 2007), boosting metabolic processes, prompting nutrient uptake, activating beneficial soil microbiota (Liu et al. 2019), and augmenting protein biosynthesis and putative hormone-like activities (Nardi et al. 2018). Additionally, HA has beneficial indirect effects, such as N loss hindrance and amelioration of soil properties such as OC pools, aggregation and stabilization of aggregates, total porosity, effective permeability, water, and nutrient-holding capacity and lowering pH levels (Imbufe et al. 2005; Gümüş and Şeker 2015; Liu et al. 2019). Several studies have documented the positive effects of HA not only on soil properties but also on plant performance by enhancing nutrient bioavailability (Khan et al. 2018; Liu et al. 2019; Mekdad et al. 2021b). However, few studies have focused on exploring the potential positive roles of HA as a soil improver in ameliorating the aggregate size distribution and other related hydro-physico-chemical properties in nutrient-deficient sandy loam soil. In this respect, Norambuena et al. (2014), Zhou et al. (2019), and Zanin et al. (2019) found that the application of HA as a soil conditioner improved the soil aggregate index by increasing soil aeration porosity and nutrient retention via metal–humic complex formation and reduced the soil bulk density.

Exogenous application of eco-friendly and cost-effective plant growth stimulants (PGSs), such as zinc oxide nanoparticles (ZnONPs) and L-tryptophan (L-TRP), to boost crop yield in normal and defective nutrient-deficient soils has recently received much attention (Jamil et al. 2018; Rizwan et al. 2019). Among the essential elements (Mekdad et al. 2021a), zinc (Zn) is an important microelement for the proper growth of plants, and its deficiency can hinder crop growth and result in yield loss (Cakmak and Kutman 2018). Moreover, Zn-deficient soils account for ~ 30% of the world’s cultivated soils, and wheat is a more sensitive crop to Zn deficiency than other cereal crops, which negatively affects yield and quality (Merchant 2010). Zn is absorbed via plant roots in a divalent cation (Zn^2+^) form. Zn is an activator of ~ 300 photosynthetic and metabolic enzymes, such as DNA- and RNA-polymerase, superoxide dismutase, and carbonic anhydrase (CA), which are involved in the photosynthesis and metabolism of carbohydrates, lipids, and nucleic acids (Singh et al. 2019). Out of the important Zn^2+^ functions, the development of the cell chloroplast acting as a cofactor of the CA enzyme enhances the chloroplast’s CO_2_ and thus increases the RuBisCO enzyme’s carboxylation capacity (Faizan et al. 2018). Zn^2+^ contributes to stomatal conductance adjustment, maintenance of biomembrane integrity, ionic homeostasis (Marschner 2012), growth regulation via biosynthesis of gibberellin, and endogenous indole-3-acetic acid (IAA) auxin (Barker and Pilbeam 2015). However, excessive Zn^2+^ supply can induce toxicity and impair wheat growth (Li et al. 2022), and plants may manifest symptoms like those observed with Cd^2+^ or Pb^2+^ exposure (Marschner 2012). On the other hand, low Zn in cereal grains, including wheat, can cause hazardous effects on human health, especially in developing African countries that highly depend on cereal-derived foods. For adults, the recommended daily intake of Zn is between 7 and 16 mg (Haase et al. 2020) because it has potent antiviral immunoregulatory effects (Read et al. 2019). In this context, Dubourg et al. (2021) suggested that Zn supplementation could be helpful for patients experiencing severe coronavirus infection.

L-tryptophan (L-TRP), chemically known as L-*β*-3-indolylalanine, is an essential amino acid not only for plants but also for humans, animals, and microorganisms (Mustafa et al. 2018). It is involved in proteosynthesis and provides the constitutional backbone for numerous secondary metabolites, such as indoleamines (i.e., melatonin and serotonin) and IAAs, in plant cells (Murch et al. 2000; Woodward and Bartel 2005). L-TRP can be applied to crop plants via different methods, such as soil application (Ul Hassan and Bano 2015), foliar spray (Al-Badrawi and Alabdulla 2021), and seed priming (Kahveci et al. 2021). Some studies have observed that L-TRP supplementation can influence cellular morphogenesis and growth and development in plants (Zahir et al. 2000). Al-Badrawi and Alabdulla (2021) noted that foliar-sprayed L-TRP at a concentration of 120 mg l^−1^ improved chlorophyll content, growth parameters, grain yield, and quality in wheat. Mohamed et al. (2018) found that exogenous foliar application of L-TRP on bread wheat crops positively affected nutrient (N, P, K, and Zn) accumulation in grains.

‏ To the best of our knowledge, no study has yet assessed the collaborative effect of soil-applied HA with exogenous foliar L-TRP or ZnONP application on bread wheat. We hypothesized that amending nutrient-poor sandy soil integrated with foliar application of ZnONPs or L-TRP would potentially improve soil hydro-physico-chemical properties and wheat yield and quality. Therefore, the main objective was to investigate the combined effect of HA and foliar L-TRP or ZnONP application on the soil hydro-physico-chemical properties, morpho-physiological parameters, grain yield, and quality of wheat grown under nutrient-poor sandy soil conditions.

## Materials and Methods

### Experimental Area and Soil Characteristics

**Fig. 1:**
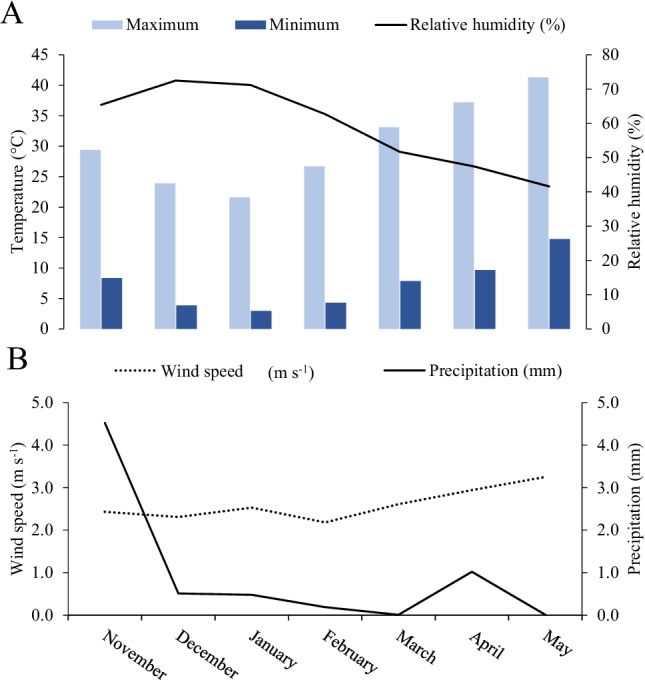
Average of monthly climatic data [maximum and minimum temperatures, and relative humidity (**A**), and wind speed and perception (**B**)] during the experiment in both 2016/2017 and 2017/2018 winter seasons in Cairo region, Egypt (data pooled over both seasons)

A 2-year (2016/17 and 2017/18) pot experiment was conducted in an open greenhouse of the Agronomy Department (latitude: 30° 3′ 16.83′′ N and longitude: 31° 19′ 11.83′′ E), Faculty of Agriculture, Al-Azhar University, Cairo, Egypt. This experiment investigated the beneficial effects of soil-applied humic acid (HA) and foliar applications of plant growth stimulants (PGSs) on the soil hydro-physico-chemical properties, morpho-physiological parameters, grain yield, and its components, and grain quality traits of wheat crops grown under nutrient-poor sandy loam soil conditions. The climate conditions of the experimental area during the experimental period of the 2016/2017 and 2017/2018 growing wheat seasons are displayed in Fig. 1. The experimental area is characterized as a semiarid environment with an average monthly day/night air temperature of 30.4 ± 2.7/7.4 ± 1.6 °C, relative humidity of 58.9 ± 4.6%, wind speed of 2.6 ± 0.1 m s^−1^, and precipitation of 1.0 ± 0.6 mm across the two-winter wheat growing seasons of this study. The soil used in this study was collected close to the experimental site at 0–0.3 m depth, and unwanted materials (i.e., litter, plant roots, stones, debris, and other coarse materials) were excluded. Before filling the pots, the soil was air-dried, ground, homogenized, and sieved through a 2-mm stainless steel sieve. The physicochemical characteristics of the tested soil were determined following standardized methods (Page et al. 1982; Klute 1986; Table 1). The soil was sandy loam textured with 49.53% coarse sand, 25.20% fine sand, 15.27% silt, and 10.0% clay contents, having a dry bulk density and total porosity of 1.54 Mg m^−3^ and 41.89%, respectively. The moisture content on a dry weight basis at field capacity (FC), permanent wilting point (PWP), and available water (AW) were 12.25%, 3.54%, and 8.71%, respectively. Chemically, this nutrient-deficient soil has pH (7.82), electrical conductivity for soil paste extract (EC_e_ = 1.55 deciSiemens per meter; dS m^−1^), OM (0.38%), SOC (0.22%), available N (20.42 mg kg^−1^), available P (5.03 mg kg^−1^), and available K (8.67 mg kg^−1^). This soil is also poor in available micronutrient concentrations, such as iron (Fe; 150.7 mg kg^−1^), Zn (4.01 mg kg^−1^), manganese (Mn; 7.12 mg kg^−1^), and copper (Cu; 2.05 mg kg^−1^).

**Table 1:** Some characteristics of the experiment soil at sowing in both 2016/2017 and 2017/2018 growing winter seasons (data pooled over both seasons)

Property	Units	Value
Coarse sand		49.53 ± 0.72
Fine sand	(%)	25.20 ± 0.46
Silt		15.27 ± 0.51
Clay		10.00 ± 0.66
Texture class		Sandy loam
Moisture content on a dry weight basis		
FC	(%)	12.25 ± 0.10
PWP		3.54 ± 0.12
AW		8.71 ± 0.18
Bulk density	(Mg m^−3^)	1.54 ± 0.04
Total porosity	(%)	41.89 ± 0.77
^*^pH		7.82 ± 0.02
^**^EC_e_	(dS m^−1^)	1.55 ± 0.03
Organic carbon	(%)	0.22 ± 0.01
Organic matter		0.38 ± 0.02
CaCO_3_		1.75 ± 0.06
^**^Soluble cations		
Ca^2+^	(mmolc l^−1^)	2.75 ± 0.03
Mg^2+^		2.50 ± 0.02
Na^+^		9.80 ± 0.08
K^+^		0.44 ± 0.05
Soluble anions		
CO_3_^2−^		–––
HCO_3_^−^	(mmolc l^−1^)	2.14 ± 0.02
Cl^−^		9.95 ± 0.62
SO_4_^2−^		3.40 ± 0.04
Available macro- and micro-nutrients		
N	(mg kg^−1^ soil)	20.42 ± 0.60
P		5.03 ± 0.07
K		8.67 ± 0.10
Fe		150.7 ± 4.2
Zn		4.01 ± 0.22
Mn		7.12 ± 0.32
Cu		2.05 ± 0.05

^(^*^)^ and ^(^**^)^ for soil analysis were measured in the suspension of soil:water 1:1 (w/v) and soil paste (1:2.5 soil:water, w/v) extract, respectively. *FC* field capacity, *PWP* permanent wilting point, *AW* available water, and *EC*_e_ electrical conductivity for soil paste extract. (*n* = 3)

### Plant Material, Treatments, Experimental Design, and Cropping Detail

Sakha 93, local high-yielding wheat (*Triticum aestivum* L.) cultivar, was kindly supplied by the Wheat Research division, Agriculture Research Center, Giza, Egypt, to be used as plant material for this experiment. The major constituents of the water dissoluble HA substance (purchased from Alpha-Chemika, Mumbai, India) are shown in Table 2. L-tryptophan (L-TRP), zinc oxide nanoparticles (ZnONPs), and other chemical reagents of analytical grade were purchased from Sigma-Aldrich (Germany) and used as received. The wheat grains were surface sterilized with a NaOCl (1%; v/v) solution for 3 min, washed twice with deionized distilled water (DDW), and air-dried. Uniform wheat-sized grains were planted in plastic pots (30 cm top inner diameter and 35 cm depth) filled with equal amounts (10 kg) of air-dried sandy loam soil that were previously described in the present study.

**Table 2:** Humic acid (HA) characteristics used in our investigation on a dry weight basis

Constituent	Concentration (%)
Pure HA	90.3
Macro- and micro-nutrients	
Nitrogen	0.94
Phosphorus	1.04
Potassium	1.46
Calcium	2.81
Magnesium	0.92
Sulfur	0.48
Iron	0.61
Manganese	0.09
Zinc	0.32
Copper	0.55
Sodium	0.04
Others	0.44

The experimental design was a 3 × 5 factorial experiment arranged in a randomized complete block design (RCBD) comprising three soil-applied HA levels (i.e., 0 (HA_0_), 0.2 (HA_0.2_), and 0.4 (HA_0.4_) g kg^−1^ soil) and five levels of foliar spraying using two PGSs [distilled water as a control, 50 mg l^−1^ zinc oxide nanoparticles (ZnONPs_50_), 100 mg l^−1^ zinc oxide nanoparticles (ZnONPs_100_), 0.25 mmol l^−1^ L-tryptophan (L-TRP_0.25_), and 0.5 mmol l^−1^ L-tryptophan (L-TRP_0.5_)] with three replicates. Each replicate consisted of three pots, totaling 135 pots for the whole experiment. Experimental pots were divided into three equal main sets, each with 45 pots, based on soil-applied HA treatments. Then, each of the former main sets was divided into five subsets, each with 15 pots, to include foliar application with different PGS treatments. Before filling the experimental pots, the used soil was thoroughly mixed well with HA treatments, urea (46% N) to fulfill the N requirements, calcium superphosphate (15.5% P_2_O_5_) to fulfill the P requirement, and K sulfate (48% K_2_O) to fulfill the K requirement. All experimental treatments were supplied with 74 kg P_2_O_5_ ha^−1^, 167 kg N ha^−1^, and 84 kg K_2_O ha^−1^ as recommended doses. The full amounts of P and K fertilizers and the first (1/3 N) dose were applied basely at planting time when filling the experimental pots, while the other second (1/3 N) and third (1/3 N) doses were applied after 25 and 50 days from planting (DFP), respectively. In each pot, eight homogenous grains were sown on November 15th and 18th in the 2016/17 and 2017/18 seasons, respectively. After 2 weeks (full emergence), five uniform seedlings were maintained per pot. The pots were irrigated regularly to maintain a proper moisture level of approximately at field capacity. Before spraying, the ZnONPs were resuspended well in DDW to increase the dispersion and solubility of their particles using an ultrasonicator (100 W; 40 kHz) instrument for 20 min. A few drops of Tween-20 were added to the foliar treatments (ZnONPs and L-TRP solutions) as an adhesive agent to increase the solution adhesion to plant leaves. A portable hand sprayer was used for spraying the different concentrations of ZnONPs and L-TRP on the upper surface of wheat leaves until there was run-off (~ 120 ml pot^−1^). Foliar applications of the different concentrations of the two PGSs (ZnONPs and L-TRP) were performed three times at three distinguished wheat growth stages (e.g., onset of tillering, onset of stem elongation, and onset of heading). These phenological stages correspond with the BBCH 20/21, BBCH 30/31, and BBCH 50/51 stages, respectively, according to the BBCH (Biologische Bundesanstalt, Bundessortenamt und CHemische Industrie) scale (Meier et al. 2009).

### Sampling and Measurements

#### Morphophysiological Parameters

At the onset of the flowering (BBCH 60/61) stage, two plants from each pot (i.e., eighteen plants per treatment) were carefully uprooted to avoid damaging the root systems of the remaining plants. These plants were accurately separated into roots and shoots (i.e., stems plus leaves), and the roots were then washed gently with tap water to remove any adhering soil particles. Root length (cm) was measured up to the tip of the longest seminal root length as described by Farrell et al. (1993) using a meter scale. Shoot length (cm) was measured from the base toward the ground to the spike tip of the main stem labeled in advance at the beginning of the tillering stage. Roots and shoots were oven-dried in a forced-air drier at 80 ± 2 °C until a constant weight was achieved to estimate their final dry weights (g) using an electronic digital balance. The leaf chlorophyll content per unit leaf area was measured from a fully extended fresh leaf using ethanol (95%; v/v) as an extractant (Lichtenthaler and Wellburn 1983). A spectrophotometer (Beckman 640D, USA) was used to measure absorbance at different wavelengths. The leaf chlorophyll a and b contents were measured at absorbance wavelengths of 665 and 649 nm, respectively, and the total leaf chlorophyll content (mg cm^−2^) was calculated by summing the chlorophyll a and b contents (Shibaeva et al. 2020).

#### Grain Yield and Its Components

The remaining three plants in each pot were harvested at the full ripening (BBCH 88/89) stage. The harvested plants were used to determine plant height (cm), spike length (cm), and spike no. plant^−1^. All spikes were oven-dried at 70 ± 2 °C until a constant weight was reached to obtain the spike dry weight pot^−1^ (g). Then, wheat grains were threshed, cleaned, and weighed to record grain weight pot^−1^ (g) (corrected to 12% grain moisture content) and 1000-grain weight (g).

#### Grain Nutrient Content and Grain Quality-Related Traits

For the determination of grain nutrient (i.e., N, P, and K in mg g^−1^ and Zn in μg g^−1^) contents based on dry weight (DW), ground and homogenized grain samples were acid-digested in a mixture of HClO_4_ and H_2_SO4 (1:3; v/v) and diluted with DDW (Jones and Benton 2001). The total N concentration was determined by the micro-Kjeldahl technique using a Kjeltec 2300 appliance (FOSS, Sweden) according to the standard method described in A.O.A. C (1990). Leaf P content (%) was colorimetrically determined by the ascorbic acid method (Watanabe and Olsen 1965) using a spectrophotometer apparatus (Beckman 640D, USA). Using a Perkin-Elmer flame photometer, the leaf K content was spectrophotometrically determined according to Chapman and Pratt (1982). Following the Higinbotham et al. (1967) procedure, the leaf Zn content was measured using a Perkin-Elmer Model 3300 Atomic Absorption Spectrophotometer apparatus. The total N concentration was multiplied by 5.7 as a converting factor to obtain the total protein content (%) in wheat grains. Wet and dry gluten (%) in the wheat grain flour were determined following a handwashing 38–10 method (AACC International 2000).

#### Soil Aggregate Size Distribution and Physicochemical Properties

At the end of the experiment, soil samples were collected from 0 to 30 cm depth using a sharpened PVC push tube with a 5-cm inner diameter. Then, these samples were well homogenized and used directly for separate soil analyses. For the dry aggregate size separation, soil samples were fully spread to air-dry on a polyethylene sheet at room temperature (~ 28 °C) in the laboratory and separated into seven different aggregate sizes (> 2, 2–1, 1–0.5, 0.5–0.25, 0.25–0.125, 0.125–0.063, < 0.063 mm) by dry-sieving and then directly weighed. The dry mean weight-diameter (MWD) was calculated according to the method described by Six et al. (2002) as follows: MWD = $\sum_{\mathrm{i}=1}^{\mathrm{n}}{\mathrm{X}}_{\mathrm{i}}{\mathrm{W}}_{\mathrm{i}}$, where *X*_i_ = mean diameter of the considered aggregate size (mm), *W*_i_ = weight percentage of the dry aggregate size class with respect to the total sample, and *n* = number of size classes. The pressure plate procedure outlined by Klute (1986) and James (1988) was gravimetrically (g/g, %) used to determine soil water retention at matric potentials of − 0.33 and − 15 bar for FC% and PWP%, respectively. Metal rings 1 cm in height and 3 cm in diameter were utilized to determine soil water retention at FC and PWP by saturating soil samples overnight with distilled water. Then, the soil samples were placed in a pressure membrane instrument at − 0.33 and − 15 bar suction pressures until the water flow stopped. The oven-dried weight of the soil samples was then determined after drying at 105 °C for 24 h. The available water (AW%) content was computed as the difference between the soil water content at FC and PWP. Total porosity (TP%) and bulk density (BD; Mg m^−3^) were determined according to Klute (1986). Soil pH was measured in a soil water (1:2.5 w/v) suspension as described in Jackson (1967). The EC_e_ (dS m^−1^) was measured using a Metler EC meter according to US Salinity Laboratory Staff (1954).

### Statistical Analysis

Before running the analysis of variance (ANOVA) for the two experimental seasons, the homogeneity of error variance (Levene 1960) for all obtained parameters was tested. The homogeneity test output indicated that all parameters were accepted for performing ANOVA. The pooled analysis of the two experimental seasons was performed based on a factorial two-way ANOVA in RCBD with two factors (i.e., HA and PGSs) as per Casella (2008) using the Genstat statistical 12th edition software statistical package (VSN Int. Ltd, Oxford, UK). Means of treatments were separated using Bonferroni’s multiple comparison test at a *p* ≤ 0.05 probability level.

## Results

### Soil Aggregate Size Distribution and Physicochemical Properties

**Fig. 2:**
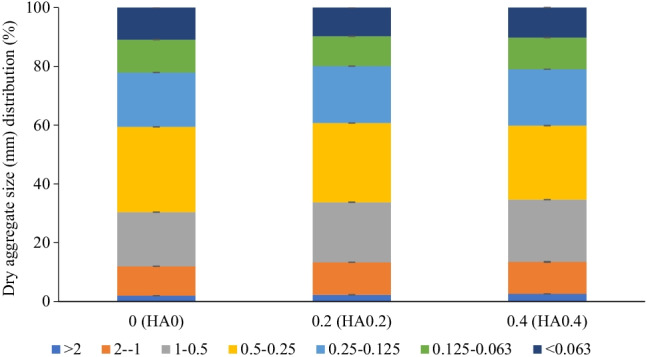
Dry aggregate size (mm) distribution (%) of the tested nutrient-poor sandy loam soil as affected by soil addition of three humic acid (HA) rates (0 (HA_0_), 0.2 (HA_0.2_), and 0.4 (HA_0.4_) g kg^−1^ soil) during 2016/2017 and 2017/2018 growing seasons (data pooled over both seasons). *p* < 0.001^**^ for 2 mm, *p* < 0.001^**^ for 2–1 mm, *p* < 0.001^**^ for 1–0.5 mm, *p* < 0.001^**^ for 0.5–0.25, *p* < 0.001^**^ for 0.25–0.125 mm, *p* < 0.001^**^ for 0.125–0.063 mm, and *p* < 0.001^**^ for < 0.063 mm, where ^(**)^ indicates to significant difference at *p* ≤ 0.01

**Fig. 3:**
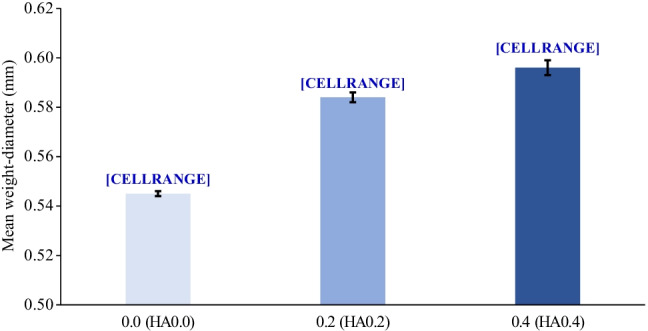
Dry mean weight-diameter (mm) of the tested nutrient-poor sandy loam soil as affected by soil addition of three humic acid (HA) rates (0 (HA_0_), 0.2 (HA_0.2_), and 0.4 (HA_0.4_) g kg^−1^ soil) during 2016/2017 and 2017/2018 growing seasons (data pooled over both seasons). *p* < 0.001^**^, where ^(**)^ indicates to significant difference at *p* ≤ 0.01. Bars sharing the same letter in each parameter are not significantly (*p* ≤ 0.05) different according to Bonferroni’s multiple comparison test

Results in Figs. 2 and 3 show that soil-applied HA_0.2_ and HA_0.4_ to the experimental sandy loam soil significantly improved the aggregate size distributions at different diameters and MWDs. Compared with HA_0_, the addition of HA_0.2_ and HA_0.4_ significantly increased the dry macroaggregates (i.e., for > 2 mm by 15.1 and 31.7%, for 2–1 mm by 8.5 and 10.6%, for 1–0.5 mm by 10.9 and 15.0%, and for 0.25–0.125 mm by 3.7 and 4.6%) and MWD by 7.2 and 9.4%; however, they decreased the dry microaggregates (i.e., for 0.125–0.063 mm by 3.6 and 9.2% and for < 0.063 mm by 6.6 and 10.5%). Soil hydro-physico-chemical properties were significantly affected by the soil-applied HA treatments (Table 3). Compared with HA_0_, the HA_0.2_ and HA_0.4_ treatments markedly increased the soil moisture content (i.e., at FC by 12.0 and 22.0%, PWP by 12.4 and 11.6%, and AW by 11.8 and 26.2%), TP by 3.9 and 7.2%, and EC_e_ by 10.9 and 16.0%, respectively. However, as the HA level increased from 0.2 to 0.4 g kg^−1^ soil, the BD and pH of the experimental soil gradually decreased from 2.6 to 5.2% and from 0.4 to 0.8%, respectively, compared with HA_0_ (Table 3).

**Table 3:** Effect of soil-applied humic acid (HA) rates on soil physico-chemical properties of the tested nutrient-poor sandy loam soil during 2016/2017 and 2017/2018 growing seasons (data pooled over both seasons)

Treatment	Soil moisture content at			TP	BD (Mg m^−3^)	pH	EC_e_ (dS m^−1^)
	FC	PWP	AW				
	(%)						
Season (S)							
2016/2017	13.64 ± 0.39a	4.15 ± 0.10a	9.49 ± 0.48a	43.0 ± 0.45a	1.51 ± 0.01a	7.81 ± 0.01a	1.70 ± 0.04a
2017/2018	13.64 ± 0.39a	4.16 ± 0.10a	9.48 ± 0.48a	42.9 ± 0.46a	1.51 ± 0.01a	7.81 ± 0.01a	1.70 ± 0.04a
HA (g kg^−1^ soil)							
0 (HA_0_)	12.25 ± 0.004c	3.54 ± 0.02b	8.71 ± 0.02c	41.5 ± 0.16c	1.55 ± 0.004a	7.84 ± 0.006a	1.56 ± 0.006c
0.2 (HA_0.2_)	13.72 ± 0.003b	3.98 ± 0.01a	9.74 ± 0.01b	43.1 ± 0.15b	1.51 ± 0.004b	7.81 ± 0.006b	1.73 ± 0.004b
0.4 (HA_0.4_)	14.94 ± 0.006a	3.95 ± 0.01a	10.99 ± 0.01a	44.5 ± 0.23a	1.47 ± 0.006c	7.78 ± 0.006c	1.81 ± 0.008a
*p* value							
S	1.00^ ns^	0.529^ ns^	0.840^ ns^	0.580^ ns^	0.580^ ns^	1.00^ ns^	0.667^ ns^
HA	< 0.001^**^	< 0.001^**^	< 0.001^**^	< 0.001^**^	< 0.001^**^	< 0.001^**^	< 0.001^**^
CV%	0.1	0.6	0.3	1.2	0.9	0.1	0.8

*FC* field capacity, *PWP* permanent welting point, *AW* available water, *TP* total porosity, *BD* bulk density, *EC*_e_ electrical conductivity for soil paste (1:2.5 soil:water, w/v) extract, and *CV%* coefficient of variation. ^(^**^)^ refers to a significant difference at *p* ≤ 0.01 and ns = not significant at *p* = 0.05. Means sharing the same letter for each factor in each column are not significantly different according to Bonferroni’s multiple comparison test. (*n* = 3)

### Wheat Morphophysiological Parameters

Compared with the unamended (HA_0_) treatment, HA_0.2_ and HA_0.4_ gradually increased wheat morphophysiological parameters, namely, shoot length by 9.4 and 12.9%, root length by 25.8 and 34.3%, shoot dry weight by 26.2 and 46.8%, root dry weight by 38.7 and 54.8%, and chlorophyll content by 18.8 and 34.0%, respectively. Among the HA × PGS interaction levels in Table 4, the interaction application of HA_0.4_ × ZnONPs_100_ followed by HA_0.4_ × L-TRP_0.50_ resulted in the highest shoot length (55.0 and 54.7 cm), root length (16.15 and 16.02 cm), shoot dry weight (6.83 and 6.62 g), root dry weight (0.60 and 0.56 g), and chlorophyll contents (59.5 and 58.9 mg cm^−2^), respectively, over both growing seasons.

**Table 4:** Effect of soil-applied humic acid (HA) rates, foliar spraying with plant growth stimulants (PGSs), and their interaction on wheat’s morpho-physiological parameters grown in a nutrient-poor sandy loam soil during 2016/2017 and 2017/2018 growing seasons (data pooled over both seasons)

Treatment		Shoot length	Root length	Shoot dry weight	Root dry weight	Chlorophyll content
		(cm)		(g)		(mg cm^−2^)
Season (S)						
2016/2017		49.0 ± 0.61a	13.15 ± 0.29a	4.66 ± 0.19a	0.41 ± 0.02a	44.6 ± 1.35a
2017/2018		49.2 ± 0.61a	13.23 ± 0.28a	4.74 ± 0.20a	0.41 ± 0.02a	44.9 ± 1.37a
HA (g kg^−1^ soil)						
0 (HA_0_)		45.7 ± 0.46c	10.99 ± 0.11c	3.78 ± 0.12c	0.31 ± 0.01c	38.2 ± 0.76c
0.2 (HA_0.2_)		50.0 ± 0.65b	13.82 ± 0.21b	4.77 ± 0.22b	0.43 ± 0.02b	45.2 ± 1.43b
0.4 (HA_0.4_)		51.6 ± 0.67a	14.76 ± 0.21a	5.55 ± 0.23a	0.48 ± 0.02a	50.9 ± 1.72a
PGSs						
Control		43.2 ± 0.44d	12.22 ± 0.42c	3.22 ± 0.10d	0.31 ± 0.01c	31.7 ± 0.43e
ZnONPs_50_		49.6 ± 0.56 b	12.62 ± 0.27b	4.24 ± 0.11c	0.40 ± 0.01b	45.8 ± 1.36c
ZnONPs_100_		52.0 ± 0.77a	14.26 ± 0.50a	5.80 ± 0.26a	0.48 ± 0.03a	51.0 ± 1.69a
L-TRP_0.25_		49.1 ± 0.50c	12.78 ± 0.29b	4.62 ± 0.25b	0.39 ± 0.02b	45.1 ± 1.33d
L-TRP_0.50_		51.6 ± 0.77a	14.08 ± 0.50a	5.62 ± 0.25a	0.47 ± 0.03a	50.3 ± 1.75b
HA × PGSs						
HA_0_	Control	40.9 ± 0.22i	9.97 ± 0.13f	2.88 ± 0.08i	0.26 ± 0.01 h	30.9 ± 0.3 k
	ZnONPs_50_	46.6 ± 0.20ef	11.17 ± 0.11e	3.75 ± 0.12e–h	0.33 ± 0.01f–h	38.5 ± 0.3 h
	ZnONPs_100_	47.7 ± 0.21e	11.42 ± 0.24e	4.42 ± 0.20de	0.32 ± 0.01f–h	42.5 ± 0.3f
	L-TRP_0.25_	46.2 ± 0.13 fg	11.17 ± 0.11e	3.60 ± 0.10gh	0.32 ± 0.02gh	37.6 ± 0.3i
	L-TRP_0.50_	47.3 ± 0.21ef	11.23 ± 0.08e	4.25 ± 0.17d–g	0.33 ± 0.01f-h	41.3 ± 0.4 g
HA_0.2_	Control	43.5 ± 0.21 h	12.65 ± 0.24d	3.07 ± 0.04hi	0.31 ± 0.01gh	30.1 ± 0.3 l
	ZnONPs_50_	50.3 ± 0.31d	12.93 ± 0.04d	4.30 ± 0.14d–f	0.43 ± 0.01de	47.0 ± 1.4e
	ZnONPs_100_	53.2 ± 0.17b	15.22 ± 0.10b	6.15 ± 0.16a–c	0.51 ± 0.02bc	50.9 ± 0.4d
	L-TRP_0.25_	50.2 ± 0.11d	13.33 ± 0.21 cd	4.33 ± 0.11d–f	0.40 ± 0.02d–f	47.3 ± 0.4e
	L-TRP_0.50_	52.9 ± 0.18bc	14.98 ± 0.12b	5.98 ± 0.13bc	0.52 ± 0.03bc	50.6 ± 0.3d
HA_0.4_	Control	45.2 ± 0.29 g	14.03 ± 0.03c	3.72 ± 0.10f–h	0.35 ± 0.01e–g	33.9 ± 0.3j
	ZnONPs_50_	52.1 ± 0.10c	13.75 ± 0.17c	4.67 ± 0.08d	0.45 ± 0.01 cd	52.0 ± 0.5c
	ZnONPs_100_	55.0 ± 0.87a	16.15 ± 0.35a	6.83 ± 0.18a	0.60 ± 0.03a	59.5 ± 0.3a
	L-TRP_0.25_	50.8 ± 0.17d	13.83 ± 0.11c	5.92 ± 0.24c	0.46 ± 0.01 cd	50.4 ± 0.4d
	L-TRP_0.50_	54.7 ± 0.24a	16.02 ± 0.10a	6.62 ± 0.8ab	0.56 ± 0.02ab	58.9 ± 0.4b
*p* value						
S		0.496^ ns^	0.423^ ns^	0.465^ ns^	0.473^ ns^	0.794^ ns^
HA		< 0.001^**^	< 0.001^**^	< 0.001^**^	< 0.001^**^	< 0.001^**^
PGSs		< 0.001^**^	< 0.001^**^	< 0.001^**^	< 0.001^**^	< 0.001^**^
HA × PGSs		< 0.001^**^	< 0.001^**^	< 0.001^**^	< 0.001^**^	< 0.001^**^
CV%		1.0	2.6	6.8	9.0	6.0

*Control* distilled water, *ZnONP*_*50*_ zinc oxide nanoparticles (50 mg l^−1^), *ZnONP*_*100*_ zinc oxide nanoparticles (100 mg l^−1^), *L-TRP*_*0.25*_ L-tryptophan (0.25 mmol l^−1^), and *TRP*_*0.50*_ L-tryptophan (0.50 mmol l^−1^). *CV%* coefficient of variation. ^(^**^)^ refers to a significant difference at *p* ≤ 0.01 and ns = not significant at *p* = 0.05. Means sharing the same letter for each factor in each column are not significantly different according to Bonferroni’s multiple comparison test. (*n* = 3)

### Wheat Grain Yield and Its Components

Data in Table 5 show that wheat grain yield and components significantly differed in response to the main effect of soil-applied HA and foliar PGSs and their interaction. Compared with the HA_0_ level, the wheat’s plant height, spike length, spike dry weight pot^−1^, grain yield pot^−1^, 1000-grain weight, and spike no. plant^−1^ under the HA_0.2_ and HA_0.4_ levels were significantly improved by 17.4 and 19.9%, 8.4 and 11.7%, 45.5 and 55.0%, 28.3 and 54.4%, 3.4 and 5.0%, and 45.2 and 57.9%, respectively. Wheat plants exogenously sprayed with ZnONPs or L-TRP showed a significant increase in their grain yield and related components compared to unsprayed control plants (Table 5). Compared with those of unsprayed control plants, higher concentrations of ZnONPs_100_ or L-TRP_0.50_ resulted in a marked increase in plant height by 22.9% and 22.9%, spike length by 59.5% and 62.8%, spike dry weight pot^−1^ by 59.1% and 63.5%, grain yield pot^−1^ by 42.4% and 47.2%, 1000-grain weight by 12.0% and 13.7%, and spike no. plant^−1^ by 104.4% and 104.4%, respectively. Results also indicated that increasing the soil-applied HA rate from HA_0_ to HA_0.2_ or HA_0.4_ combined with foliar spraying with ZnONPs or L-TRP strongly affected wheat grain yield and its components. HA_0.4_ × ZnONPs_100_ followed by HA_0.4_ × L-TRP_0.50_ exhibited the highest plant height (77.0 and 77.3 cm), spike length (12.10 and 12.18 cm), spike dry weight pot^−1^ (22.58 and 23.00 g), grain yield pot^−1^ (17.25 and 18.15 g), 1000-grain weight (48.0 and 48.9 g), and spike no. plant^−1^ (6.33 and 7.00), respectively (Table 5).

**Table 5:** Effect of soil-applied humic acid (HA) rates, foliar spraying with plant growth stimulants (PGSs), and their interaction on grain yield and its components of wheat grown in a nutrient-poor sandy loam soil during 2016/2017 and 2017/2018 growing seasons (data pooled over both seasons)

Treatment		Plant height	Spike length	Spike dry weight pot^−1^	Grain yield pot^−1^	1000-grain weight	Spike no. plant^−1^
		(cm)		(g)			
Season (S)							
2016/2017		69.3 ± 1.1b	10.59 ± 0.26a	16.87 ± 0.63a	12.45 ± 0.42a	45.5 ± 0.3a	4.91 ± 0.22a
2017/2018		69.8 ± 1.2a	10.64 ± 0.27a	16.99 ± 0.64a	12.53 ± 0.42a	45.6 ± 0.5a	4.84 ± 0.23a
HA (g kg^−1^ soil)							
0 (HA_0_)		61.9 ± 1.1c	9.95 ± 0.34c	12.68 ± 0.32c	9.79 ± 0.21c	44.3 ± 0.3c	3.63 ± 0.18c
0.2 (HA_0.2_)		72.7 ± 0.9b	10.79 ± 0.33b	18.45 ± 0.63b	12.56 ± 0.26b	45.8 ± 0.4b	5.27 ± 0.23b
0.4 (HA_0.4_)		74.2 ± 0.9a	11.11 ± 0.28a	19.66 ± 0.61a	15.12 ± 0.46a	46.5 ± 0.4a	5.73 ± 0.24a
PGSs							
Control		59.3 ± 1.6d	7.26 ± 0.18d	12.07 ± 0.44e	9.74 ± 0.38d	41.7 ± 0.1d	2.72 ± 0.16b
ZnONPs_50_		71.8 ± 1.4b	11.04 ± 0.12c	16.11 ± 0.72d	12.15 ± 0.42c	45.9 ± 0.2c	5.28 ± 0.24a
ZnONPs_100_		72.9 ± 1.2a	11.58 ± 0.15b	19.20 ± 0.94b	13.87 ± 0.67b	46.7 ± 0.3b	5.56 ± 0.23a
L-TRP_0.25_		71.0 ± 1.4c	11.37 ± 0.08b	17.53 ± 0.65c	12.34 ± 0.48c	45.9 ± 0.2c	5.28 ± 0.25a
L-TRP_0.50_		72.9 ± 1.2a	11.82 ± 0.11a	19.74 ± 0.98a	14.34 ± 0.73a	47.4 ± 0.4a	5.56 ± 0.33a
HA × PGSs							
HA_0_	Control	50.4 ± 0.2i	6.38 ± 0.15 h	9.72 ± 0.16 h	7.65 ± 0.15i	41.3 ± 0.2 h	2.00 ± 0.22e
	ZnONPs_50_	63.8 ± 0.3gh	10.42 ± 0.08e	11.92 ± 0.20 g	9.98 ± 0.11 h	44.9 ± 0.1ef	4.00 ± 0.26c
	ZnONPs_100_	66.4 ± 0.4e	10.80 ± 0.12de	13.78 ± 0.18f	10.50 ± 0.17gh	45.2 ± 0.1ef	4.33 ± 0.21c
	L-TRP_0.25_	62.9 ± 0.3 h	10.97 ± 0.11d	13.92 ± 0.08f	10.03 ± 0.03 h	44.7 ± 0.1f	3.83 ± 0.17 cd
	L-TRP_0.50_	66.0 ± 0.6ef	11.18 ± 0.07 cd	14.08 ± 0.05f	10.78 ± 0.11 fg	45.4 ± 0.1e	4.00 ± 0.26c
HA_0.2_	Control	62.8 ± 0.3 h	7.25 ± 0.09 g	12.45 ± 0.23 g	10.33 ± 0.21gh	41.7 ± 0.1gh	2.83 ± 0.17de
	ZnONPs_50_	75.3 ± 0.2 cd	11.22 ± 0.05 cd	18.02 ± 0.07e	12.32 ± 0.17e	46.1 ± 0.1d	5.83 ± 0.17b
	ZnONPs_100_	75.5 ± 0.3b–d	11.83 ± 0.13ab	21.23 ± 0.12c	13.87 ± 0.12d	47.0 ± 0.1c	6.00 ± 0.23b
	L-TRP_0.25_	74.3 ± 0.2d	11.55 ± 0.04bc	18.40 ± 0.16e	12.20 ± 0.09e	46.2 ± 0.1d	6.00 ± 0.20b
	L-TRP_0.50_	75.6 ± 0.2b–d	12.10 ± 0.06a	22.15 ± 0.16b	14.10 ± 0.05 cd	47.9 ± 0.2b	5.67 ± 0.21b
HA_0.4_	Control	64.6 ± 0.5 fg	8.15 ± 0.07f	14.05 ± 0.05f	11.23 ± 0.12f	42.1 ± 0.1 g	3.33 ± 0.21 cd
	ZnONPs_50_	76.2 ± 0.5a–c	11.50 ± 0.05bc	18.39 ± 0.14e	14.15 ± 0.11 cd	46.7 ± 0.1 cd	6.00 ± 0.01ab
	ZnONPs_100_	77.0 ± 0.5ab	12.10 ± 0.11a	22.58 ± 0.46ab	17.25 ± 0.40b	48.0 ± 0.3b	6.33 ± 0.17ab
	L-TRP_0.25_	75.8 ± 0.3a–d	11.60 ± 0.04bc	20.27 ± 0.19d	14.80 ± 0.16c	46.9 ± 0.1c	6.00 ± 0.01ab
	L-TRP_0.50_	77.3 ± 0.3a	12.18 ± 0.05a	23.00 ± 0.03a	18.15 ± 0.09a	48.9 ± 0.1a	7.00 ± 0.26a
*p* value							
S		0.030^ ns^	0.297^ ns^	0.451^ ns^	0.658^ ns^	0.287^ ns^	0.225^ ns^
HA		< 0.001^**^	< 0.001^**^	< 0.001^**^	< 0.001^**^	< 0.001^**^	< 0.001^**^
PGSs		< 0.001^**^	< 0.001^**^	< 0.001^**^	< 0.001^**^	< 0.001^**^	< 0.001^**^
HA × PGSs		< 0.001^**^	< 0.001^**^	< 0.001^**^	< 0.001^**^	< 0.001^**^	0.001^**^
CV%		1.0	2.0	2.1	2.6	0.6	9.7

*Control* distilled water, *ZnONP*_*50*_ zinc oxide nanoparticles (50 mg l^−1^), *ZnONP*_*100*_ zinc oxide nanoparticles (100 mg l^−1^), *L-TRP*_*0.25*_ L-tryptophan (0.25 mmol l^−1^), and *TRP*_*0.50*_ L-tryptophan (0.50 mmol l^−1^). *CV%* coefficient of variation. ^(^**^)^ refers to a significant difference at *p* ≤ 0.01 and *ns* = not significant at *p* = 0.05. Means sharing the same letter for each factor in each column are not significantly different according to the Bonferroni’s multiple comparison test. (*n* = 3)

### Grain Nutrient Content and Grain Quality-Related Traits

Data presented in Table 6 show that amendments with HA_0.2_ and HA_0.4_ resulted in significant increases in grain nutrients (i.e., N by 10.5 and 13.1%, P by 8.3 and 16.3%, K by 10.5 and 13.3%, and Zn by 11.8 and 26.5%), total protein in grains by 10.2 and 13.4%, wet gluten by 18.2 and 23.3%, and dry gluten by 23.5 and 29.5%, respectively, compared with the unamended (HA_0_) treatment. The uptake and accumulation of grain nutrients and grain quality-related traits gradually increased with increasing ZnONP or L-TRP supplementation compared with the unsprayed control. L-TRP_0.50_ markedly increased grain nutrients (i.e., N by 22.6%, P by 39.5%, and K by 23.7%), total protein in grains by 21.6%, wet gluten by 15.8%, and dry gluten by 22.6%, while Zn content in wheat grains increased by 157.1% due to ZnONPs_100_ supplementation over the unsprayed control plants. Regarding the HA × PGS interactions, the highest contents of N (20.0 mg g^−1^ DW), P (4.72 mg g^−1^ DW), K (23.6 mg g^−1^ DW), total protein (11.38%), and wet (32.3%) and dry (12.34%) gluten in grains were observed in wheat plants treated with the HA_0.4_ × ZnONPs_100_ interaction, closely followed by those treated with the HA_0.4_ × L-TRP_0.50_ interaction (Table 6). The highest Zn content (0.062 μg g^−1^DW) in grains was obtained when applying the HA_0.4_ × ZnONPs_100_ interaction.

**Table 6:** Effect of soil-applied humic acid (HA) rates, foliar spraying with plant growth stimulants (PGSs), and their interaction on grain nutrient content and grain quality-related traits of wheat grown in a nutrient-poor sandy loam soil during 2016/2017 and 2017/2018 growing seasons (data pooled over both seasons)

Treatment		Nitrogen	Phosphorus	Potassium	Zinc (μg g^−1^ dry weight)	Total protein	Wet gluten	Dry gluten
		(mg g^−1^ dry weight)				(%)		
Season (S)								
2016/2017		17.5 ± 0.22a	3.63 ± 0.07b	19.8 ± 0.26b	0.038 ± 0.002b	9.92 ± 0.14a	26.9 ± 0.43a	9.78 ± 0.19a
2017/2018		17.4 ± 0.25b	3.66 ± 0.07a	20.3 ± 0.29a	0.039 ± 0.002a	9.93 ± 0.14a	27.1 ± 0.44a	9.85 ± 0.20a
HA (g kg^−1^ soil)								
0 (HA_0_)		16.2 ± 0.19c	3.37 ± 0.05c	18.6 ± 0.20c	0.034 ± 0.002c	9.20 ± 0.12c	23.7 ± 0.09c	8.34 ± 0.07c
0.2 (HA_0.2_)		17.9 ± 0.24b	3.65 ± 0.07b	20.5 ± 0.29b	0.038 ± 0.002b	10.14 ± 0.14b	28.0 ± 0.21b	10.30 ± 0.11b
0.4 (HA_0.4_)		18.3 ± 0.27a	3.92 ± 0.10a	21.0 ± 0.32a	0.043 ± 0.002a	10.43 ± 0.16a	29.2 ± 0.47a	10.80 ± 0.21a
PGSs								
Control		15.3 ± 0.21d	3.01 ± 0.02e	17.8 ± 0.22e	0.021 ± 0.001e	8.77 ± 0.13d	24.6 ± 0.31d	8.81 ± 0.16d
ZnONPs_50_		17.3 ± 0.17c	3.52 ± 0.06d	19.6 ± 0.18d	0.046 ± 0.001b	9.87 ± 0.10c	26.9 ± 0.55c	9.65 ± 0.26c
ZnONPs_100_		18.3 ± 0.31b	3.82 ± 0.06b	20.8 ± 0.30b	0.054 ± 0.002a	10.42 ± 0.19b	28.1 ± 0.70b	10.22 ± 0.31b
L-TRP_0.25_		17.4 ± 0.14c	3.69 ± 0.06c	20.0 ± 0.20c	0.033 ± 0.001d	9.91 ± 0.09c	26.8 ± 0.57c	9.59 ± 0.26c
L-TRP_0.50_		18.8 ± 0.32a	4.20 ± 0.11a	22.0 ± 0.44a	0.038 ± 0.001c	10.66 ± 0.19a	28.5 ± 0.82a	10.80 ± 0.34a
HA × PGSs								
HA_0_	Control	14.2 ± 0.80i	2.93 ± 0.30i	16.5 ± 0.07 l	0.018 ± 0.001 l	8.09 ± 0.05j	23.0 ± 0.18i	7.93 ± 0.06i
	ZnONPs_50_	16.4 ± 0.02 g	3.27 ± 0.04 h	18.7 ± 0.15ij	0.040 ± 0.001f	9.36 ± 0.02 h	23.8 ± 0.07gh	8.20 ± 0.02i
	ZnONPs_100_	16.6 ± 0.04 g	3.58 ± 0.09 fg	19.1 ± 0.06 h	0.045 ± 0.002d	9.43 ± 0.02 h	24.2 ± 0.04 g	8.51 ± 0.12 h
	L-TRP_0.25_	16.6 ± 0.03 fg	3.42 ± 0.05gh	19.0 ± 0.11hi	0.031 ± 0.001i	9.48 ± 0.02 h	23.5 ± 0.03 h	8.10 ± 0.01i
	L-TRP_0.50_	17.0 ± 0.04f	3.65 ± 0.60d–f	19.5 ± 0.08 g	0.034 ± 0.003 h	9.67 ± 0.02 g	24.1 ± 0.03 g	8.98 ± 0.07 g
HA_0.2_	Control	15.8 ± 0.14 h	3.07 ± 0.03i	18.3 ± 0.08 k	0.020 ± 0.001 k	9.06 ± 0.03i	26.0 ± 0.07e	9.46 ± 0.06f
	ZnONPs_50_	17.6 ± 0.08e	3.52 ± 0.05 fg	19.8 ± 0.12 g	0.046 ± 0.002d	10.00 ± 0.03f	28.0 ± 0.02d	10.19 ± 0.08e
	ZnONPs_100_	19.0 ± 0.03c	3.78 ± 0.03d	21.4 ± 0.09d	0.056 ± 0.003b	10.75 ± 0 .02d	29.0 ± 0.08c	10.62 ± 0.14d
	L-TRP_0.25_	17.6 ± 0.07e	3.67 ± 0.04d-f	20.3 ± 0.18f	0.031 ± 0.002i	10.00 ± 0.02f	27.9 ± 0.04d	10.16 ± 0.08e
	L-TRP_0.50_	19.3 ± 0.10bc	4.23 ± 0.08b	21.9 ± 0.26c	0.037 ± 0.001 g	10.92 ± 0.02c	29.1 ± 0.12c	11.08 ± 0.07c
HA_0.4_	Control	15.9 ± 0.28 h	3.03 ± 0.04i	18.5 ± 0.07jk	0.024 ± 0.003j	9.17 ± 0.02i	24.9 ± 0.08f	9.05 ± 0.09 g
	ZnONPs_50_	18.0 ± 0.10d	3.77 ± 0.05de	20.3 ± 0.15f	0.052 ± 0.002c	10.24 ± 0.06e	29.0 ± 0.05c	10.56 ± 0.10d
	ZnONPs_100_	19.5 ± 0.26b	4.10 ± 0.08bc	22.8 ± 0.18b	0.062 ± 0.005a	11.09 ± 0.16b	31.0 ± 0.35b	11.54 ± 0.15b
	L-TRP_0.25_	18.0 ± 0.08d	3.98 ± 0.07c	20.8 ± 0.10e	0.037 ± 0.001 g	10.26 ± 0.05e	28.9 ± 0.09c	10.51 ± 0.09d
	L-TRP_0.50_	20.0 ± 0.03a	4.72 ± 0.05a	23.6 ± 0.33a	0.042 ± 0.001e	11.38 ± 0.02a	32.3 ± 0.08a	12.34 ± 0.11a
*p* value								
S		0.033^*^	0.038^*^	0.018^*^	0.007^*^	0.439^ ns^	0.422^ ns^	0.151^ ns^
HA		< 0.001^**^	< 0.001^**^	< 0.001^**^	< 0.001^**^	< 0.001^**^	< 0.001^**^	< 0.001^**^
PGSs		< 0.001^**^	< 0.001^**^	< 0.001^**^	< 0.001^**^	< 0.001^**^	< 0.001^**^	< 0.001^**^
HA × PGSs		< 0.001^**^	< 0.001^**^	< 0.001^**^	< 0.001^**^	< 0.001^**^	< 0.001^**^	< 0.001^**^
CV%		1.0	2.5	0.9	2.7	0.8	0.8	1.4

*Control* distilled water, *ZnONP*_*50*_ zinc oxide nanoparticles (50 mg l^−1^), *ZnONP*_*100*_ zinc oxide nanoparticles (100 mg l^−1^), *L-TRP*_*0.25*_ L-tryptophan (0.25 mmol l^−1^), and *TRP*_*0.50*_ L-tryptophan (0.50 mmol l^−1^). *CV%* coefficient of variation. ^(^*^)^ and ^(^**^)^ refer to a significant difference at *p* ≤ 0.05 and *p* ≤ 0.01, respectively, ns = not significant at *p* = 0.05. Means sharing the same letter for each factor in each column are not significantly different according to the Bonferroni’s multiple comparison test. (*n* = 3)

### Correlation Analysis Among Soil and Wheat Crop Parameters

**Fig. 4:**
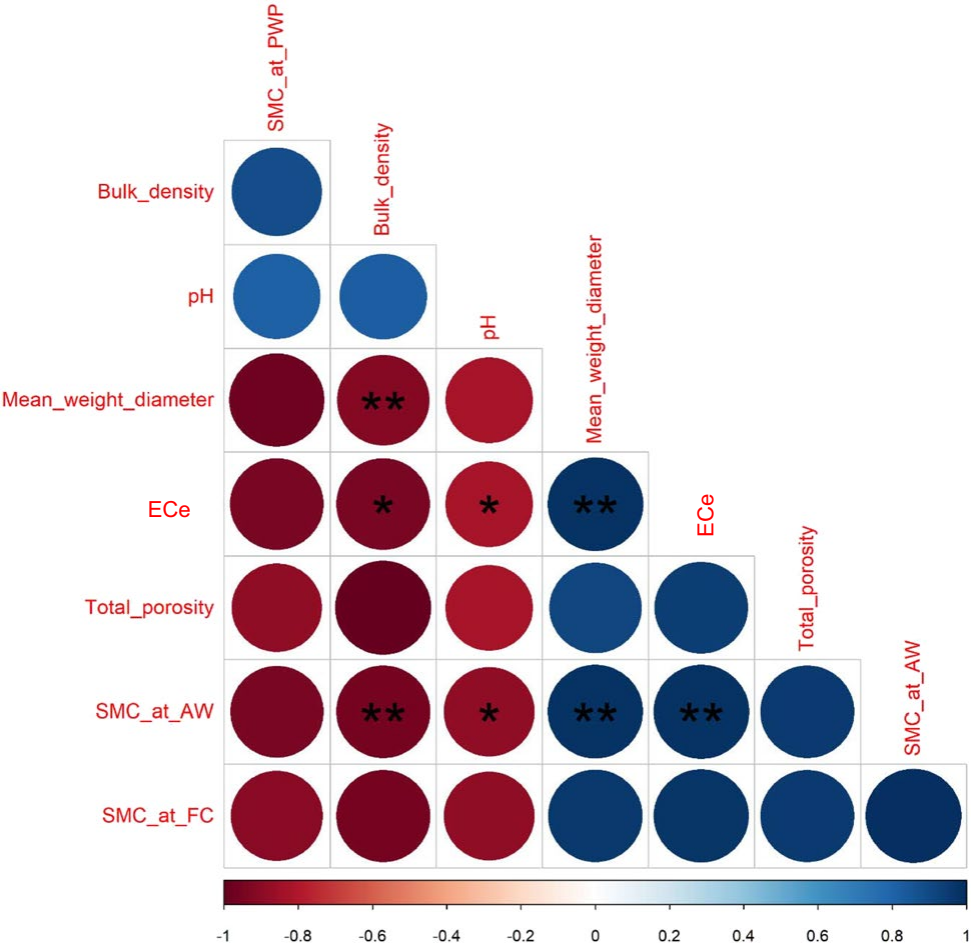
Heatmap of simple Pearson’s correlation coefficients matrix of the soil parameters, i.e., dry mean weight-diameter (MWD), soil moisture content (SMC) at field capacity (FC%), permanent wilting point (PWP%), and available water (AW%), total porosity (TP%), bulk density (BD; Mg m^−3^), and electrical conductivity for soil paste extract (ECe; dS m^−1^) as affected by soil addition of three humic acid (HA) rates (0 (HA_0_), 0.2 (HA_0.2_), and 0.4 (HA_0.4_) g kg^−1^ soil) during 2016/2017 and 2017/2018 growing seasons (data pooled over both seasons). ^(^*^)^ and ^(^**^)^ refer to significant correlations at *p* ≤ 0.05 and *p* ≤ 0.01, respectively

**Fig. 5:**
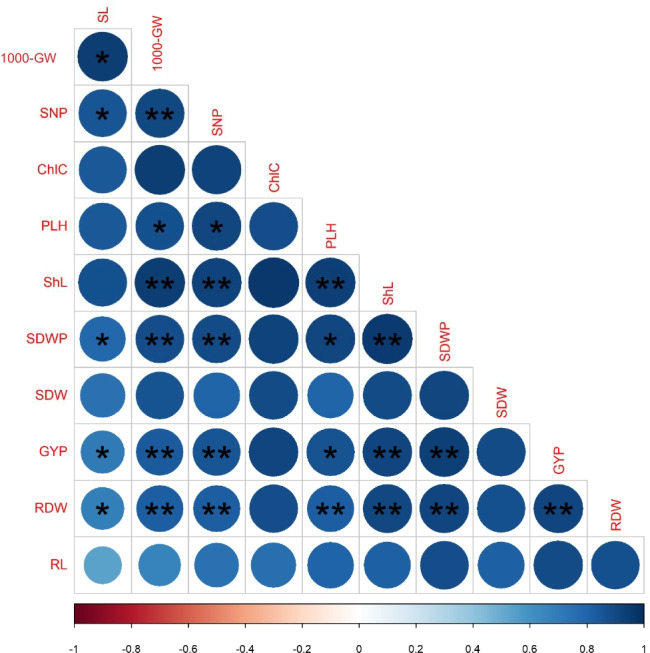
Heatmap of simple Pearson’s correlation coefficients matrix of wheat parameters, i.e., shoot length (ShL; cm), root length (RL; cm), shoot dry weight (SDW; g), root dry weight (RDW; g), leaf chlorophyll content (ChlC; mg cm^−2^), plant height (PLH; cm), spike length (SL; cm), spike dry weight pot^−1^ (SDWP; g), grain weight pot^−1^ (GYP; g), 1000-grain weight (1000-GW; g), and spike no. plant^−1^ (SNP) as affected by the interactive of soil addition of three humic acid (HA) rates (0 (HA_0_), 0.2 (HA_0.2_), and 0.4 (HA_0.4_) g kg^−1^ soil) and five levels of PGSs, included zinc oxide nanoparticles (ZnONPs) and L-tryptophan (L-TRP) [control, 50 mg l^−1^ (ZnONPs50), 100 mg l^−1^ (ZnONPs_100_), 0.25 mmol l^−1^ (L-TRP_0.25_), and 0.5 mmol l^−1^ (L-TRP_0.5_)] during 2016/2017 and 2017/2018 growing seasons (data pooled over both seasons). ^(^*^)^ and ^(^**^)^ refer to significant correlations at *p* ≤ 0.05 and *p* ≤ 0.01, respectively

To explore the interrelationship among the measured traits at the soil and plant levels, correlation analysis was performed on the data obtained under our experimental conditions. For the soil measurements from the data obtained under HA treatments over both seasons, Fig. 4 shows that soil BD was significantly (*p* ≤ 0.01) negatively correlated with MWD (− 0.92^**^), TP (1.0), and moisture content at FC (− 0.95^**^), and AW (− 0.95^**^), whereas TP was significantly (*p* ≤ 0.01) positively correlated with MWD (0.92), moisture content at FC (0.95^**^), and AW (0.95^**^). For the wheat plant measurements from the data obtained under the HA × PGS interaction over both seasons, Fig. 5 shows that grain yield pot − ^1^ and 1000-grain weight were significantly (*p* ≤ 0.01) positively correlated with shoot length (0.91^**^ and 0.95^**^), root length (0.90^**^ and 0.67^**^), shoot dry weight (0.90^**^ and 0.86^**^), root dry weight (0.91^**^ and 0.82^**^), chlorophyll content (0.92^**^ and 0.94^**^), plant height (0.87^**^ and 0.88^**^), spike length (0.70^**^ and 0.94^**^), spike dry weight pot − ^1^ (0.93^**^ and 0.89^**^), and spike no. plant − ^1^ (0.85^**^ and 90^**^), respectively.

## Discussion

Our experimental soil is classified as sandy loam and is characterized by a relatively high pH, low EC_e_, low OM content, OC content, and nutrient deficiency. These unfavorable characteristics make it defective and less productive because it is classified as having poor hydro-physico-chemical properties. Osman (2018) reported that the management of nutrient-deficient sandy soils poses several challenges. Among these challenges are high hydraulic conductivity, infiltration rate, gas permeability, specific heat, pH level, and sensitivity to compaction but low WHC, low CEC, low concentrations of OM and OC, weak structure and fertility, nutrient leaching and agrochemical runoff, and nutritional imbalance (Yost and Hartemink 2019). To maintain crop productivity grown in nutrient-deficient sandy soil, amending it with organic fertilizers such as HA (Zhou et al. 2019) and supplying its crop plants with PGSs such as ZnONPs or L-TRP are needed, especially for dryland agriculture.

In nutrient-poor sandy soil, a good structure is critical for increasing crop productivity and improving environmental quality, and it is employed as a soil status indicator (Yazdanpanah et al. 2016). The formation of macroaggregates in the studied soil due to HA addition increased via the coactive binding of microaggregates (Regelink et al. 2015) during the experimental period. These findings are consistent with those of Zhou et al. (2019), who found that adding a bentonite-HA mixture to sandy soil increased the ratio of macroaggregate sizes (> 2 mm) while decreasing the ratio of microaggregate sizes (0.25 mm). The lack of binding materials such as clay is the primary cause behind the poor structure of sandy loam soil. However, the application of HA, an organic material, to nutrient-poor sandy soil boosts soil structure stabilization by cementing soil particles for clay-organic compound formation (Regelink et al. 2015), which increases MWD and improves soil aggregate stability. The increase in macroaggregate ratios may be related to HA application, which decreased BD and increased TP and soil moisture at FC and AW, thereby enhancing wheat root growth. Roots play a vital role in increasing macroaggregates by acting as a binding agent (Miao et al. 2017), and hence, increased root growth likely encouraged the genesis of macroaggregates under HA_0.2_ or HA_0.4_ relative to HA_0_ treatment. HA addition to sandy loam soil can enhance the soil water content to be slowly released as crops need by adsorption onto electrically charged compounds and organo-mineral complexes in the soil that correlate with swelling complexes (Zhou et al. 2019). Additionally, Qin and Leskovar (2020) and Zhang et al. (2018) found a significant correlation between increasing soil OC and water productivity due to the formation of water-stable macroaggregates under soil humic substance application. Our data showed that the addition of HA_0.2_ or HA_0.4_ to soil significantly resulted in a slight and significant, respectively, increase in EC_e_ and decrease in pH level, which is in line with Ding et al. (2021) for HA application. These changes could be due to the high CEC of HA due to its active functional groups, such as -COO, -OH, and -NH_2_ (Wang et al. 2019), nutrient contents, and active organic acids.

The wheat morphophysiological parameters were significantly boosted by soil HA supplementation. Comparable observations were reported by Zanin et al. (2019) and Nasiroleslami et al. (2021), who found that root and shoot growth traits and leaf chlorophyll content showed a higher response to HA application in wheat. This might be due to improved soil characteristics, particularly those related to soil moisture retention, soil aeration porosity, bioavailability and uptake of macro- and micro-nutrients, and soil microbial activity that positively reflected root length, photosynthetic and physiological parameters, and ultimately growth and productivity (Liu et al. 2019; Zhou et al. 2019; Ding et al. 2021).

Sandy loam soils usually suffer from rapid leaching losses of many nutrients, especially Zn, because of their high mobility in sandy loam soils and their weak binding to their particles (Shaaban et al. 2022). Therefore, foliar supplementation with ZnONPs and L-TRP plays a beneficial role in improving crop plant tolerance to several abiotic stresses and under low soil supply conditions (Jamil et al. 2018; Rizwan et al. 2019). In the current study, foliar ZnONP or L-TRP application significantly improved wheat morphophysiological parameters, which were due to support of cell division and enlargement and remobilization of carbohydrate reserves to plants under sandy loam soil conditions.

Foliar application of ZnONPs_100_ or L-TRP_0.50_ markedly increased wheat morpho-physiological and grain yield-related parameters compared with the control. A similar result regarding the positive effects of ZnONPs on wheat was found by Hussain et al. (2018) and Adrees et al. (2021), who observed that foliar application of ZnONPs caused a significant increase in growth, total chlorophyll content, and yield and related parameters of wheat. The increase in growth and leaf chlorophyll content in ZnONP treatment in our study is because Zn is reported to play a pivotal role as a structural and catalytic component of proteins and metabolic-related enzymes and as a cofactor required for stimulating the expression of chlorophyll biosynthesis genes (Rastogi et al. 2017). It upregulated the activity of important Zn-dependent enzymes such as CA and fructose 1,6 diphosphatase, which stimulate CO_2_ hydration, facilitating CO_2_ diffusion to carboxylation sites (Faizan et al. 2018), resulting in better growth, grain yield, and related attributes. Regarding the positive effects of L-TRP on morphophysiological responses and grain yield components, Al-Badrawi and Alabdulla (2021) observed better wheat growth, chlorophyll content, grain yield, and its components after foliar application of L-TRP at different concentrations compared to the control treatment. This may be attributed to the increase in internodal elongation and leaf area resulting from the growth-stimulatory effects of L-TRP, which mediated an essential role in the biosynthesis of phytohormones, mainly IAA and gibberellins (Mustafa et al. 2018).

The wheat grain yield, its components, and grain nutrients (e.g., N, P, K, and Zn), total grain protein, and wet and dry gluten content of grains were significantly improved with HA_0.2_ or HA_0.4_ addition compared to HA_0_. Improvements in wheat yield-related traits and grain quality by HA addition were observed in several previous studies (Khan et al. 2018; Nasiroleslami et al. 2021). Additionally, Dinçsoy and Sönmez (2019) found that soil application of HA considerably improved the nutritional (i.e., P, K, and Zn) content of wheat grains. The bioavailability of soil nutrients for wheat plants in our defective sandy loam soil might be achieved by increasing OM and OC content through HA application. This positive result is also due to the improvement of structure stability, aeration porosity, and water retention of soil and the reduction in soil pH, which is positively reflected in the increase in the nutrient content of wheat grains, grain yield, and quality in terms of protein and gluten (Dinçsoy and Sönmez 2019; Zhou et al. 2019; Nasiroleslami et al. 2021).

Foliar spraying with ZnONPs_100_ and L-TRP_0.50_ markedly increased the grain nutrient content, yield-related traits, and grain quality of bread wheat compared to the control. These positive effects in our study are in accordance with those of Hussain et al. (2018) and Adrees et al. (2021) on wheat and faba bean crops. The improvement in grain yield under ZnONP supplementation may be because Zn causes the allotment of more nutrients to the sink (i.e., reproductive) organs and boosts higher dry matter accretion (Amanullah and Inamullah 2016). Furthermore, the results of L-TRP on yield parameters of wheat were in accordance with Ul Hassan and Bano (2015), Mohamed et al. (2018), and Al-Badrawi and Alabdulla (2021), who found better wheat grain yield and its components after foliar application with L-TRP in comparison to the control treatment. The reason behind the higher grain nutrients, grain yield, and content of protein and gluten in the grains of L-TRP-treated wheat could be due to the positive effect of L-TRP on growth, chlorophyll biosynthesis, and canopy gas exchange parameters such as the CO_2_ assimilation rate and stomatal conductance, resulting in a higher net photosynthetic rate and dry matter accumulation (Kahveci et al. 2021). The optimum results for most parameters of the soil and wheat crops in this study were obtained through the interaction of the high rate (i.e., 0.4 g kg^−1^ soil) of soil-applied HA with foliar application of any of the PGSs at a high concentration (i.e., ZnONPs_100_ or L-TRP_0.50_). These positive results for the soil and wheat crop were obtained with the benefits of the combined application of HA to the soil with exogenous plant growth promoter supplementation under nutrient-deficient sandy loam soil conditions.

## Conclusion

The addition of humic acid improved the soil hydro-physico-chemical properties of the degraded nutrient-poor sandy soil. As an excellent soil amendment, humic acid improved the soil structure by allowing rapid macroaggregate formation, decreasing bulk density and pH, and increasing total soil porosity, electrical conductivity, dry mean weight-diameter, and soil water retention capacity. Improving the hydro-physico-chemical properties of degraded nutrient-poor sandy soil positively reflected morphophysiological responses, grain yield, and grain quality. Exogenous foliar application of zinc oxide nanoparticles or L-tryptophan, especially at higher concentrations compared to the control, had a positive effect on wheat morpho-physiological responses, which consequently boosted grain yield and quality. In conclusion, applying humic acid as a soil amendment in combination with foliar application of zinc oxide nanoparticles or L-tryptophan can be used for improving the yield and quality of wheat grown on degraded nutrient-poor sandy soil under semiarid conditions. Further studies are required to investigate the potential effects of higher levels of humic acid and zinc oxide nanoparticles/L-tryptophan than levels tested in the present study. These high levels suggested for prospective studies may have more potential positive effects on soil properties and plant performance than our present studied levels; however, the economic cost aspects should be considered.

## Data Availability

All data and materials included in this work are available.
